# Hyperinsulinemic Hypoglycemia Due to *PMM2* Mutation in Two Siblings with Autosomal Recessive Polycystic Kidney Disease

**DOI:** 10.3390/pediatric14040052

**Published:** 2022-10-24

**Authors:** Ratna Acharya, Kiran Upadhyay

**Affiliations:** 1 Division of Pediatrics, University of Florida, Gainesville, FL 32610, USA; 2 Department of Pediatrics, Division of Pediatric Nephrology, University of Florida, Gainesville, FL 32610, USA

**Keywords:** PMM2, ARPKD, hyperinsulinemia, hypoglycemia

## Abstract

**Background**: Hyperinsulinemic hypoglycemia (HH) is an important cause of persistent hypoglycemia in newborns and infants. Recently, *PMM2* (phosphomannomutase 2) mutation has been associated with HH, especially in conjunction with polycystic kidney disease (PKD). *PMM2* deficiency is one of the most common causes of congenital disorder of glycosylation (CDG). Renal involvement in PMM2-CDG manifests as cystic kidney disease, echogenic kidneys, nephrotic syndrome or mild proteinuria. **Case Summary**: Here, we describe a pair of siblings with HH associated with autosomal recessive polycystic kidney disease (ARPKD) and *PMM2* mutation. Two siblings with ARPKD presented during infancy and early toddler years with severe hypoglycemia. Both had inappropriately elevated serum insulin, low β-hydroxybutyrate, a need for a high glucose infusion rate, positive glycemic response to glucagon, positive diazoxide response and *PMM2* mutation. **Conclusions**: Although this combination of HH and PKD was recently described in patients of European descent who also had *PMM2* mutation, our report is unique given that these non-consanguineous siblings were not exclusively of European descent. *PMM2* mutation leading to abnormal glycosylation and causing cystic kidneys and the alteration of insulin secretion is the most likely pathogenesis of this clinical spectrum.

## 1. Introduction

Hyperinsulinemic hypoglycemia (HH) is the most common cause of persistent and recurrent neonatal hypoglycemia [1]. It could be primary due to alteration in genes such as *HK1*, *ABCC8*, *KCNJ11*, *HNFA4*, *PGM1*, *PMM2*, *CACNA1D*, *FOXA2* and *EIF2S3* genes, among others [2]. The secondary causes of HH include congenital syndromes, intra-uterine growth restriction, maternal diabetes, perinatal asphyxia and following gastrointestinal surgery [2]. PMM2 (phosphomannomutase 2) deficiency, the most common autosomal recessive congenital disorder of glycosylation (CDG), also called carbohydrate-deficient glycoprotein syndrome or CDG type 1a, also has been recently shown to be associated with HH [3,4].

Autosomal recessive polycystic kidney disease (ARPKD) is characterized by enlarged kidneys and congenital hepatic fibrosis and presents primarily in infancy and childhood. It is caused by mutations in one of the largest human genes, *PKHD1* (polycystic kidney and hepatic disease 1), located on chromosome 6p12 [5]. *PKHD1* encodes fibrocystin which is a single-membrane spanning protein with a long extracellular N-terminus and short cytoplasmic C-terminus, and it is expressed predominantly in the apical membranes and primary cilia/basal body of kidney (collecting ducts and thick ascending limb of loop of Henle), liver (bile ducts) and pancreas [5,6]. Besides the most common renal and hepatic manifestations, a few reports of ARPKD associated with fibrocystic pancreatic changes or intracranial aneurysms have also been described [7,8]. Recently, Cabezas et al. reported the role of *PMM2* in renal morphogenesis by showing a promoter mutation in *PMM2* in 17 patients of European descent with hyperinsulinemia and polycystic kidney disease (PKD) presenting with asymptomatic cysts to end stage renal disease [3].

Here, we describe a co-occurrence of the HH and ARPKD in two siblings from non-consanguineous parents of non-European descent. 

## 2. Case Presentation

A 13-month-old toddler was evaluated with renal sonogram for ARPKD due to his younger brother recently being diagnosed with ARPKD and hypertension at 6 weeks of life. His sonogram showed bilaterally enlarged diffusely echogenic kidneys (measuring 9 cm and 9.5 cm in length) with multiple tiny anechoic cysts consistent with ARPKD. Hepatic sonogram also showed tiny anechoic cystic structures within the homogenous appearing parenchyma without evidence of fibrotic changes. Genetic testing confirmed the *PKHD1* mutation. Renal and hepatic function tests were normal. Blood pressure was 110/60 mm Hg. He was born at term via cesarean section with a birth weight of 3.9 kg. He required a one week stay in the neonatal intensive care unit for hypoglycemia. There was no history of gestational diabetes, respiratory distress or oligohydramnios. Physical examination was normal except for palpable kidneys. His repeat blood glucose levels were 68 mg/dL and 41 mg/dL when checked at two different occasions during infancy, but no further interventions were deemed necessary at that time. 

Family history showed that he lived with his younger brother, four-year old adopted sister and his parents. The maternal grandfather was from Puerto Rico and had no known history of kidney disease. The maternal grandmother had an ancestry from Norway/Germany and had no history of kidney, pancreatic or liver disease. The paternal grandfather and grandmother both were from the Dominican Republic. None of them had known kidney or liver diseases. Besides the thalassemia trait in the father, the parents were otherwise healthy and were not related to each other. There was no family history of seizure, diabetes or hypoglycemia. 

He presented at 18 months of age with a seizure episode associated with accompanying hypoglycemia with blood glucose of 30 mg/dL. He had a history of excessive snacking and frequently looking for food. He also appeared very weak after waking up in the mornings. Investigations showed an inappropriately elevated serum insulin level of 7.5 mcunit/mL with the serum glucose of 30 mg/dL. Other tests showed C-peptide 1.61 ng/mL (normal 0.5–2 ng/mL), β-hydroxybutyrate 0.2 mmol/L (normal < 0.4–0.5 mmol/L), growth hormone 0.28 ng/mL, and serum cortisol 7 mcg/dL with ACTH stimulation peak of 22.2 mcg/dL. He had a positive glucagon stimulation test (>30 mg/dL increase in serum glucose within 10 min), normal serum ammonia, serum amino acids and urine organic acids. Renal function test showed normal serum electrolytes and serum creatinine of 0.3 mg/dL with normal serum transaminases. Urinalysis showed no proteinuria, dipstick negative for blood and urine glucose ≥ 500 mg/dL with absence of ketones. Thyroid function test was normal. Head computed tomography and electroencephalograms were normal. He needed glucose infusion with glucose infusion rate (GIR) of 8 mg/kg/min to prevent hypoglycemia, and then started on high dose diazoxide (12 mg/kg/day). Despite being on the diazoxide, he continued to have intermittent glucose levels in the 60 s. Given partial but suboptimal response to the diazoxide therapy, a 12 h safety fast was performed to determine the overall response to diazoxide therapy. He did not pass the safety fast, as his serum glucose dropped below 50 mg/dL nine hours into the fast. He was then continued on GIR 3.5 mg/kg/min, as well as diazoxide 8 mg/kg/day. Diuril was also started at 12 mg/kg/day twice daily due to edema caused by diazoxide along with mild hypertension. Due to inappropriately low serum β-hydroxybutyrate and inappropriately elevated serum insulin, congenital hyperinsulinism was suspected. Genetic testing for congenital hyperinsulinism panel showed no pathogenic variants in *ABCC8, GCK, GLUD1, KCNJ11, HADH, HNF1A, HNF4A, SLC16A1* and *UCP2* genes by sequencing or deletion/duplication analysis. CDG testing via *PMM2* gene sequencing (Prevention Genetics, Marshfield, WI, USA) showed that the patient was heterozygous in the *PMM2* gene for a sequence variant designated c.368G>A, which is predicted to result in an amino acid substitution p.Arg123Gln and known to be pathogenic for CDG type 1a (CDG-1a) [9]. The patient was also heterozygous in the *PMM2* gene for a pre-coding variant designated c.-167G>T, known to be pathogenic [9]. He was discharged home on diazoxide and diuril with stable serum glucose and blood pressures. 

His younger brother who had been diagnosed with ARPKD (confirmed with *PKHD1* mutation) and hypertension at the age of 6 weeks also presented with asymptomatic hypoglycemia at 9 months of age. His birth history was unremarkable with birth weight of 3.1 kg and had no perinatal complications. His physical examination was also normal except for palpable kidneys. He presented with similar laboratory findings, clinical course and outcome as his elder brother. Based on his glucose needs, frequent hypoglycemia within 3 h after a feed, glucagon response and response to diazoxide, hyperinsulinism was suspected. Genetic testing was also exact similar as his brother. He was discharged home on diazoxide and enalapril.

### Follow-Up

The two siblings are now 6 and 7 years old with still stable renal function and hepatic enzymes. Renal sonograms have shown continued enlarged bilateral kidneys with numerous tiny cysts along with heterogenous echotexture of liver with multiple hepatic cysts and patent portal vein with hepatopetal flow. Serum glucose have been well controlled on current diazoxide therapy. 

## 3. Discussion

HH is commonly associated with mutations in *ABCC8* and *KCNJ11* genes which are involved in the regulation of insulin release from pancreatic β cells [1]. Cabezas et al. reported HH in patients with mutation in the *PMM2* gene without mutations in *ABCC8* and *KCNJ11* genes [3]. The same report described a single nucleotide variant (c.-167G > T) in the promoter region of *PMM2*, both as homozygous and compound heterozygous, with a pathogenic coding variant in trans in 17 patients from 11 unrelated families of European descent who had both HH and PKD [3]. Islam et al. also reported high-density genotyping data from 11 patients from seven unrelated families and identified a common haplotype that included the promoter variant. All these patients who were of European descent shared a 0.312 Mb haplotype which was absent in 503 European controls, with the estimated age of mutation of 105–110 generations [9]. Dorval et al. identified a pathogenic *PMM2* variant at position-167 in the coding exons in three European families with six patients with cystic kidney disease; hypoglycemia was reported in one case [10]. Moreno Macian F et al. also described four unrelated Spanish families with PKD and HH [11] Muller et al. reported a 5-year-old boy with HH with hypertension secondary to ARPKD who was treated with nifedipine with normalization of blood pressures and glucose [12]. A genetic testing was not obtained.

PMM2-CDG, a type I CDG, is a multisystem inborn error of metabolism and is due to defect of protein N-glycosylation. The clinical spectrum is broad manifesting mainly as developmental delay, hypotonia, neurological abnormalities, cardiac and gastrointestinal disorders, skin manifestations, endocrine dysfunction, hepatopathy and coagulopathy [13]. Hypoglycemia is a common manifestation. A systematic review of the literature of PMM2-CDG patients by Moravej et al. reported a 2.5% incidence of hypoglycemia; less than half of those patients had hyperinsulinism, and 70% of patients with hyperinsulinism was successfully treated with diazoxide [14] Schiff et al. presented a review of 96 patients with PMM2-CDG with the mean age of diagnosis of 6.8 years. The most common presenting signs were neurological including hypotonia, intellectual disability or cerebellar syndrome. About 50% of patients had Arg141His *PMM2* variant [4]. Renal involvement is rare in PMM2-CDG [13] Altassan et al. reported renal involvement in 56 out of 933 patients with confirmed *PMM2* deficiency [13]. The most common findings were cystic kidney and mild proteinuria; six children had congenital nephrotic syndrome. Nineteen patients had cystic kidneys (detected either by sonogram, biopsy and/or autopsy). Among the patients with cystic kidneys, most of the patients either died in utero or reported to be of toddler age group [13]. Most of the renal findings were reported to occur early in infancy. Bilateral renal echogenicity on sonogram is also not uncommon [15]. Strom et al. described five patients with the CDG syndrome who all had abnormal renal structure, two patients had multiple renal microcysts on autopsy [16]. Macian et al. reported a heterozygote variant c.-167G > T in the promoter region of *PMM2* in six patients from four unrelated Spanish families with PKD and HH [11]. All of these patients also carried a compound heterozygote for a second missense mutation in *PMM2* (p.Arg141His, p.Asp148Asn or p.Phe157Ser), pathogenic for CDG type 1a, with an AR inheritance pattern [11]. Soares et al. also described a 6-year-old female with similar association [17]. Our patients were heterozygous in the *PMM2* gene for a sequence variant c.368G > A, which has been reported to be causative for CDG-Ia [18] They were also heterozygous in the *PMM2* gene for a pre-coding sequence variant c.167G > T, known to cause disease in 17 patients from 11 European families who presented with HH and PKD [3]. In vitro data suggest that this particular variant impairs binding of the transcriptional activator ZNF143 and decreases *PMM2* transcription. Additionally, this variant is absent from large population databases of presumably healthy individuals [19,20]. 

The pathogenesis of CDG is due to defective synthesis of asparagine (N)-linked glycans, resulting in abnormal protein and lipid glycosylation. Glycosylation is the post-translational addition of glycans of proteins and lipids. The malfunctioning *PMM2* gene, located on chromosome 16p13.3–p13.2, leads to the production of abnormal PMM2 enzyme, which is involved in the conversion of mannose-6-phosphate into mannose-1-phosphate, leading to deficiency of guanosine diphosphate (GDP)-mannose. Deficiency of GDP-mannose leads to hypoglycosylation of glycoproteins such as serum proteins, lysosomal enzymes and membrane glycoproteins [21]. Abnormalities in N-glycosylation leads to disturbed metabolism, cell recognition, cell adhesion, host defense, cell migration and antigenicity. Type I CDG occurs due to the defects in the assembly or transfer of the dolichol-linked glycan in either the cytosol or the endoplasmic reticulum [18]. In N-glycosylation, N-linked glycans are attached to the asparagine residue on the protein. With regard to kidney, glycosylation is an important step in the biogenesis and function of many membrane proteins which are involved in the development of fetal kidney. Many membrane proteins are glycoproteins including the heavily N-glycosylated polycystin 1 (TRPP1, transient receptor potential channel) and polycystin 2 (TRPP2). These proteins are involved in multiple signaling pathways in an order to maintain normal renal tubular structure and function. Defective glycosylation can alter the function of both glycoproteins and lead to cystic formation in autosomal dominant PKD [22]. Inoue et al. showed that aberrant glycosylation and localization of polycystin 1 causes polycystic kidney in an AQP11 knockout model [23]. Hence, impaired glycosylation due to *PMM2* mutation may be responsible for pathogenesis of ARPKD. Mutations in sulfonylurea receptor (SUR) due to defective glycosylation has been shown to be involved in HH [24]. This highlights the importance of proper glycosylation for normal kidney development and insulin secretion. Defective glycosylation secondary to *PMM2* mutation may explain the clinical phenotype described in this report. What makes our report unique is that the two siblings affected are not exclusively of European descent as described in prior reports. 

In this report, the index of suspicion of HH was high due to elevated plasma insulin levels in the context of hypoglycemia. Both patients had enlarged kidneys with numerous cysts along with hepatic cysts; however, their renal and hepatic function remained stable until 6 and 7 years of age, respectively during the most recent follow-up. Hypertension was well controlled in both siblings with antihypertensive agents. Both the brothers had same mutations in *PMM2*. 

Most patients with HH secondary to *PMM2* variants are large for gestational age and have early onset hypoglycemia. The hypoglycemia is usually diazoxide-responsive [3]. Diazoxide binds to the SUR1 subunit of intact ATP-sensitive potassium channels in the beta cells of pancreas causing hyperpolarization of cell and inhibition of insulin release. It has also been used in the treatment of hyperinsulinemia secondary to refractory insulinomas, or in those who are poor candidates for surgery, and also in those with congenital HH [25].

## 4. Conclusions

The concurrent presence of HH in these two siblings with ARPKD associated with *PMM2* promoter mutation suggests a role of glycosylation in this relatively novel clinical phenotype. It remains to be studied whether *PMM2* mutation is responsible for the outcome in ARPKD or accelerates the pathogenesis of ARPKD. Overall, this study provides a potential link between *PMM2* mutation and ARPKD, which encourages future studies to further investigate the potential interlink between *PKHD1* and *PMM2* genes.

## Data Availability

Not applicable.
